# A new technique to monitor conidia of aquatic hyphomycetes in streams using latex-coated slides

**DOI:** 10.1080/21501203.2015.1110209

**Published:** 2015-11-07

**Authors:** Sudeep D. Ghate, Kandikere R. Sridhar

**Affiliations:** Department of Biosciences, Mangalore University, Mangalagangotri, Mangalore574 199, India

**Keywords:** conidial attachment, diversity, freshwater hyphomycetes, suspended conidia, technique

## Abstract

We examined the pattern of adherence of aquatic hyphomycetes conidia on six latex-coated slides (*Artocarpus heterophyllus, A. hirsutus, Calotropis gigantea, Ficus benghalensis, Manilkara zapota* and *Plumeria rubra*) with plain slides (control) exposed up to 18 h in a tropical coastal stream. Conidia of 21 species were trapped on latex-coated slides against seven on control slides. The total conidia adhered on latex-coated slides was higher than control slides. Latex-coated slides showed the highest diversity of aquatic hyphomycetes than control slides (1.805) and water (0.729). The top five species of aquatic hyphomycetes in latex-coated slides and drift conidia were comparable. Sørensen’s similarity of species in control slides against latex-coated slides ranged from 25% (*P. rubra*) to 62.5% (*C. gigantea*) indicating superiority of latex-coated slides in conidial trapping. Among the latex-coated slides, similarity varied between 13.3% (*A. hirsutus* vs. *P. rubra*) and 89.6% (*F. benghalensis* vs. *M. zapota*). One-way ANOVA showed significant difference in richness of species (*P* < 0.001) and conidia (*P* < 0.05) between control and latex-coated slides by *F. benghalensis*. Based on the trapping efficiency of species and conidia, the latex of *F. benghalensis* ranked first and serves as an inexpensive technique to monitor aquatic hyphomycetes in streams.

## 1. Introduction

Aquatic hyphomycetes are the dominant mycota of freshwater streams throughout the world involved in detritus decomposition and energy flow to the higher tropic levels (Bärlocher and Kendrick 1974; Suberkropp and Klug 1976; Bärlocher 1992; Gessner and Chauvet 1994). Aquatic hyphomycetes are conventionally identified based on their conidial morphology as they produce characteristic sigmoid and multiradiate conidia as an adaptation to dispersal in flowing waters similar to plankton (Webster and Davey 1984; Webster 1987; Sridhar 2009). One of the fundamental mechanisms of function of aquatic hyphomycetes is the adherence of their conidia to available solid surfaces in aquatic ecosystems (Webster and Davey 1984; Dang et al. 2007; Kearns and Bärlocher 2008). A series of events such as conidial adherence, germination/appressoria formation, colonization, release of conidia and their dispersal are cyclic in streams (Read et al. 1992; Au et al. 1996; Sridhar 2005; Dang et al. 2007). As aquatic hyphomycetes invest more than 50% of their biomass for formation of conidia, the entrapment of their conidia to new substrates for their nutrition and perpetuation in streams is crucial (Suberkropp 1991; Chauvet and Suberkropp 1998; Sridhar and Bärlocher 2000). Colonization of leaves by aquatic hyphomycetes in streams will be augmented by various mechanisms and their colonization through conidia is most predominant (Sridhar and Bärlocher 1997). Various techniques have been employed to assess aquatic hyphomycete communities’ structure and function in freshwater streams (e.g. detritus incubation, water filtration and foam assessment) (Iqbal and Webster 1973; Descals 2005; Graça et al. 2005). Briefly, stream-derived detritus rinsed to remove debris followed by submerged incubation (in Petri dishes) or bubble-chamber incubation (in conical flasks and aeration through Pasteur pipette) induces conidia of aquatic hyphomycetes. Filtering stream water through Millipore filters (5–8 μm) traps drift conidia of aquatic hyphomycetes. As aquatic hyphomycete conidia are trapped in stream foam, its microscopic examination also helps to study their community. Bärlocher et al. (1977) developed another interesting method to assess the colonization of aquatic hyphomycetes on rosin-coated slides (rosin is a non-steam-distillable component of pine resin) in streams and suggested it as a simple method for fast evaluation. Plant latex-coated on microscopic slides served as substratum as well as nutrient source for the growth and sporulation of selected aquatic hyphomycetes (Sridhar and Kaveriappa 1987). Therefore, we hypothesized that latex-coated slides also efficiently trap conidia of aquatic hyphomycetes in streams. Thus, the purpose of this study was to examine the diversity and efficiency of adherence of conidia on slides coated with different latexes in comparison with control (plain slides) and drift conidia. It is expected that trapping conidia of aquatic hyphomycetes on latex-coated slides in streams serve as a new technique to complement leaf litter, water and foam assessment.

## 2. Materials and methods

### 2.1. Stream

Experiment on the adherence of conidia of aquatic hyphomycetes on latex-coated slides was carried out during southwest monsoon (15–18 September 2014). The second-order coastal stream (Konaje) was chosen for the study. The stream passing through the laterite terrain of Mangalore University Campus followed by an arboretum and mixed plantations is located about 5 km from the Arabian Sea (12°48′N, 74°55′E; 90 m asl).

### 2.2. Latex smears

Latex from six common plant species in and around Konaje stream was selected for coating slides. Three of them were shrubs [*Calotropis gigantea* (L.) W.T. Alton (Apocyanaceae), *Manilkara zapota* (L.) P. Royen (Sapotaceae) and *Plumeria rubra* L. (Apocyanaceae) and the remaining three were tree species [*Artocarpus heterophyllus* Lam. (Moraceae), *A. hirsutus* Lam. (Moraceae) and *Ficus benghalensis* L. (Moraceae)]. Latex was collected by cutting petioles of mature green leaves. One or two drops of latex oozing from petiole was collected on sterile glass slide and smeared with another sterile slide like blood smear preparation. The smears were allowed to dry at laboratory temperature (27.5–29.5 °C) up to 12 h before immersing in stream.

### 2.3. Preliminary study

Prior to initiate the experiment, a trial was performed by immersing series of latex-coated slides in the stream up to 24 h. Latex-coated slides were vertically mounted side by side in grooves of thermocol sheet and placed in perforated plastic boxes. The boxes were immersed by tying to tree trunks/roots and their position was set in stream to expose latex-coat to face flowing water. At every 6 h intervals, slides were retrieved from stream, stained with aniline blue (0.1%) in lactophenol by applying cover glass and preserved in slide boxes. Microscopic examination (Nikon YS100, Nikon Corporation, Tokyo) revealed that conidial attachment was low during 6–12 h, moderate during 18 h without overlap (a few conidia showed germ tube initiation and formation of appressoria without losing identity) and conidia almost overcrowded at 24 h (with extended germination and dense growth masking the identity). Besides, above 18 h exposure, latex-coated slides accumulated more sediment/debris which interfered with microscopic examination. Thus, 18 h exposure of latex-coated slides in stream was chosen for conidial adherence study.

### 2.4. Conidial adherence

Latex-coated slides were prepared in the evening and dried until next day and immersed in the stream at about 12 noon. In each perforated plastic box, seven slides were fixed to thermocol consisting one each of control and six different latex-coated slides. Likewise five boxes were prepared and exposed in Konaje stream in five sites at a distance of 20 m. The experiment ended after 18 h by collecting stream-exposed slides followed by their fixation in aniline blue lactophenol and mounting with cover glass (40 × 22 mm). Conidia adhered below the cover glass area (880 mm^2^) were considered for identification and enumeration by light microscopic examination. Based on the conidial morphology and comparison with monographs aquatic hyphomycete species were identified (Ingold 1975; Nawawi 1985; Marvanová 1997; Santos-flores and Betancourt-Lopez 1997).

### 2.5. Abiotic factors

After starting the experiment at 12 noon, at every 6 h up to 18 h abiotic factors (air and water parameters) were assessed at five sites where boxes were immersed. Air humidity and temperature (in shade during day) were measured 1 m above the water surface using digital thermohygrometer (Mextech Digital Thermohygrometer, Mumbai). Water temperature was measured by mercury thermometer (N.S. Dimple Thermometer, New Delhi). The pH and conductivity of stream water were assessed by water analyser (Systronics, Ahmedabad). Water samples were fixed on the sampling site, brought to the laboratory and dissolved oxygen assessed using the Winkler’s method (APHA 1995).

### 2.6. Conidial drift

To evaluate conidial drift of aquatic hyphomycetes during the study period, water samples (100 ml in 25 ml aliquots) were collected from the sites where boxes were immersed, filtered (Millipore filters: porosity 5 μm; diam., 25 mm) and fixed with 1% lactophenol aniline blue at every 6 h interval up to 18 h. Later, filters were mounted with lactic acid and scanned using a light microscope for assessment of conidia of aquatic hyphomycetes.

### 2.7. Data analysis

The diversity of aquatic hyphomycete conidia adhered on control and latex-coated slides was evaluated by Shannon’s diversity (Magurran 1988) and Pielou’s equitability (Pielou 1975). Sørensen’s similarity indexes (%) were calculated to assess similarity of aquatic hyphomycete communities through conidia adhered on control and latex-coated slides (Chao et al. 2005). One-way ANOVA and multiple comparisons were carried out by Tukey’s test to find out relationship between richness of species and conidia among different latexes and control (SigmaPlot, Version # 11, Systat Inc., USA).

## 3. Results

### 3.1. Abiotic factors

Air temperature during experimental period was lower than stream water temperature (27.1 vs. 28.3°C) with high humidity (87.8%) (Table 1). The pH of water was almost neutral (7.1) with 90.2 µS cm^−1^ conductivity and 6.9 mg l^−1^ dissolved oxygen.

**Table 1.** Air and water parameters measured in Konaje stream during aquatic hyphomycetes conidial trap experiment (*n* = 20, mean ± SD; range in parenthesis).

Air		Water			
Temperature (°C)	Humidity (%)	Temperature (°C)	pH	Conductivity (µS cm^−1^)	Dissolved oxygen (mg l^−1^)
27.1 ± 0.21	87.8 ± 2.1	28.3 ± 0.36	7.1 ± 0.21	90.2 ± 18.2	6.9 ± 0.15
(26.6–27.3)	(85–90)	(27.5–28.5)	(6.7–7.36)	(70.4–118)	(6.8–7.2)

### 3.2. Conidial drift

Filtering stream water samples during the experimental period resulted in occurrence of 10 species of aquatic hyphomycetes with highest score of *Lunulospora curvula* followed by *Anguillospora longissima, Flagellospora curvula* and *Triscelophorus monosporus* (Table 2). The average number of species and conidia in water were 4.8 and 34 per 100 ml, respectively.

**Table 2.** Average drift conidia of aquatic hyphomycetes in 100 ml of Konaje stream water during conidial trap experiment (*n* = 20).

	Average conidia (100 ml^−1^)	Conidial contribution (%)
*Lunulospora curvula* Ingold	13.2	38.8
*Anguillospora longissima* (Sacc. & P. Syd.) Ingold	8.8	25.8
*Flagellospora curvula* Ingold	7.6	22.3
*Triscelophorus monosporus* Ingold	1.2	3.5
*Triscelophorus konajensis* K.R. Sridhar & Kaver.	0.8	2.4
*Cylindrocarpon* sp.	0.8	2.4
*Clavariopsis aquatica* De Wild.	0.4	1.2
*Dwayaangam cornuta* Descals	0.4	1.2
*Flagellospora* sp.	0.4	1.2
*Phalangispora constricta* Nawawi & J. Webster	0.4	1.2

### 3.3. Conidial adherence

**Figure 1.**
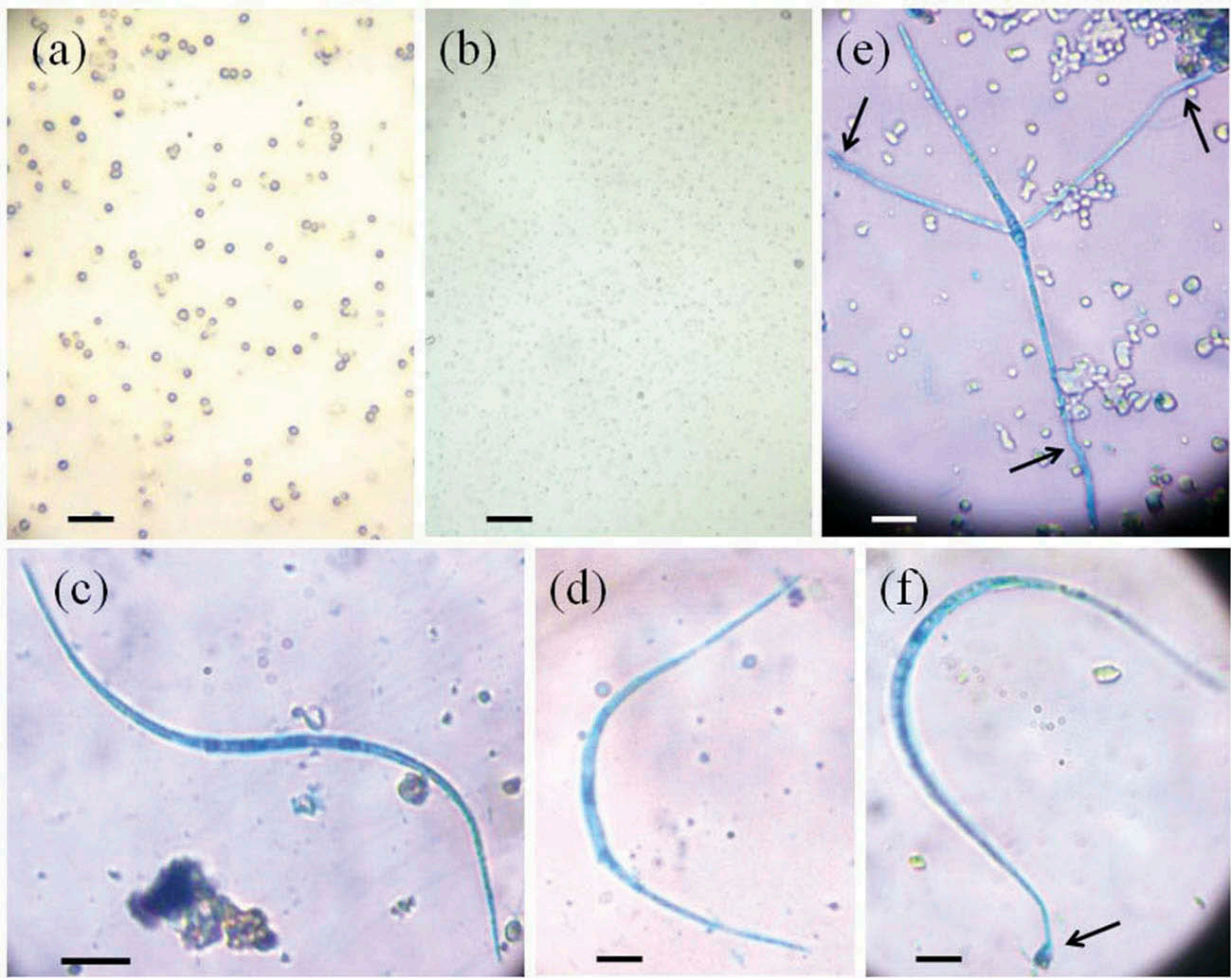
Latex-coat of banyan (*Ficus benghalensis*) (a) and jack (*Artocarpus heterophyllus*) (b) on glass slides; entrapped conidium of *Anguillospora longissima* (c), *Lunulospora curvula* (d) and *Triscelophorus monosporus* (arrows: germ tubes) (e) on banyan latex stained by aniline blue in lactophenol; Entrapped conidium of *L. curvula* on jack latex stained by aniline blue in lactophenol showing appressorium (arrow) (f) (scale bar, 10 μm).

Conidial adherence experiment on control and latex-coated slides resulted in occurrence of 22 species (Table 3). The *L*. *curvula* ranked first (and found in control and all latexes used) followed by *T. monosporus, A. longissima* and *F. curvula*. Adherence of conidia on the latex-coated slides was clear and some of them showed appressoria and germ tubes (Figure 1).

**Table 3.** Average conidia of aquatic hyphomycetes adhered on plain slides (CONT) and on latex-coated slides of six plant species exposed in Konaje stream for 18 h (ARHE, *Artocarpus heterophyllus*; ARHI, *A. hirsutus*; CAGI, *Calotropis gigantea*; FIBE, *Ficus benghalensis*; MAZA, *Manilkara zapota*; PLRU, *Plumeria rubra*) (area: 40 × 22 mm; *n* = 5).

Aquatic hyphomycete	Latex						
	CONT	ARHE	ARHI	CAGI	FIBE	MAZA	PLRU
*Lunulospora curvula* Ingold	3.0	30.8	3.2	4.2	39.0	9.2	0.2
*Triscelophorus monosporus* Ingold	0.4	7.8	0.4	3.6	27.8	3.0	–
*Anguillospora longissima* (Sacc. & P. Syd.) Ingold	–	1.2	1.2	3.2	5.6	4.8	–
*Flagellospora curvula* Ingold	0.4	5.0	0.8	2.2	3.2	1.0	–
*Triscelophorus konajensis* K.R. Sridhar & Kaver.	–	1.0	–	1.2	5.4	1.0	0.6
*Lambdasporium* sp.	–	0.2	–	0.6	1.8	–	2.0
*Flagellospora* sp.	0.2	1.0	–	0.4	0.6	0.4	0.4
*Triscelophorus acuminatus* Nawawi	–	1.2	–	–	0.6	0.4	–
*Anguillospora crassa* Ingold	–	–	–	–	0.2	0.2	1.6
*Cylindrocarpon* sp.	0.2	–	–	0.6	0.8	0.2	–
*Trisulcosporium* sp.	–	0.4	–	–	1.0	0.2	–
*Flagellospora penicillioides* Ingold	–	–	–	–	1.0	0.6	–
*Dwayaangam cornuta* Descals	–	–	–	–	–	–	1.4
*Alatospora acuminata* Ingold	–	–	–	–	0.4	0.2	0.6
Unidentified sp. (tri-radiate conidia)	0.2	0.2	0.2	–	0.2	0.2	–
*Clavatospora tentacula* Sv. Nilsson	–	–	0.2	–	0.4	–	–
*Condylospora spumigena* Nawawi	–	0.2	–	0.2	–	–	–
*Diplocladiella scalaroides* G. Arnaud	–	0.2	–	–	–	–	–
*Isthmotricladia gombakiensis* Nawawi	–	–	–	–	–	0.2	–
*Phalangispora constricta* Nawawi & J. Webster	–	–	–	–	–	–	0.2
*Clavariopsis aquatica* De Wild.	–	–	–	–	–	–	0.2
*Titaea complexa* K. Matsush. & Matsush.	0.2	–	–	–	–	–	–

**Figure 2.**
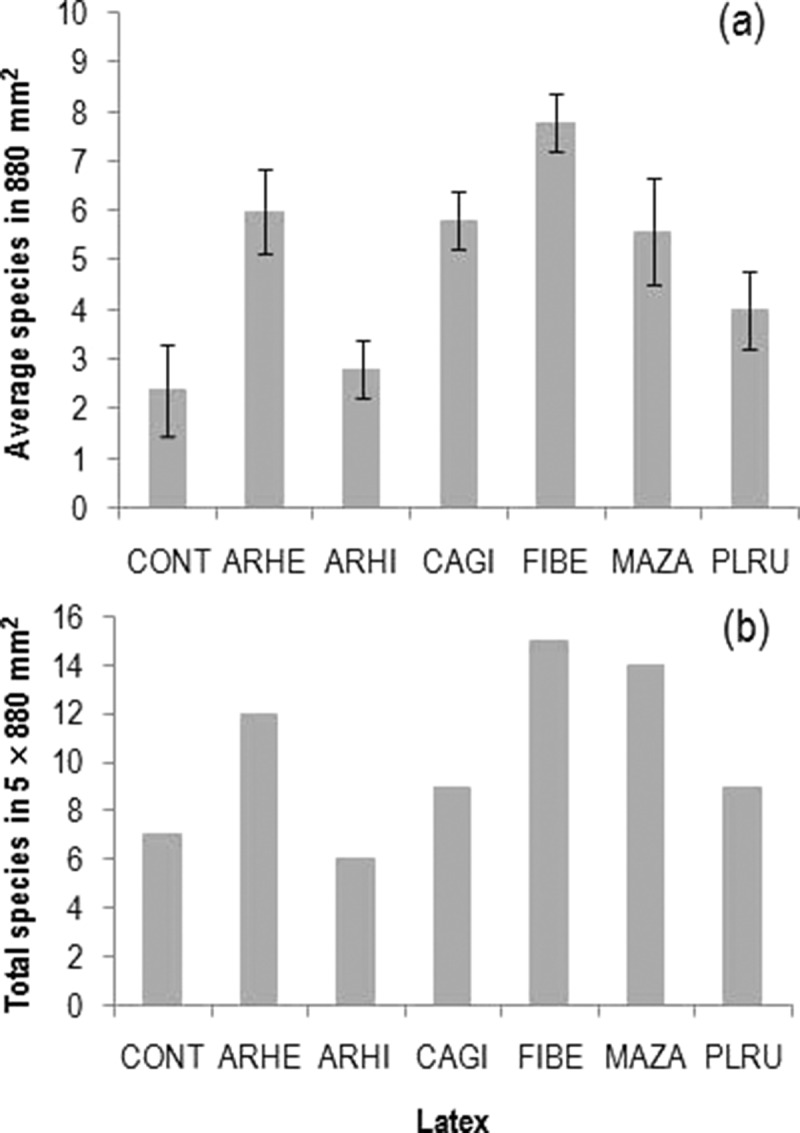
Average species (in 880 mm^2^) (*n* = 5, mean ± SE) (a) and total species (in 5 × 880 mm^2^) (b) of aquatic hyphomycetes adhered on six latexes in comparison with control (CONT) (latex code: ARHE, *Artocarpus heterophyllus*; ARHI, *A. hirsutus*; CAGI, *Calotropis gigantea*; FIBE, *Ficus benghalensis*; MAZA, *Manilkara zapota*; PLRU, *Plumeria rubra*).

**Figure 3.**
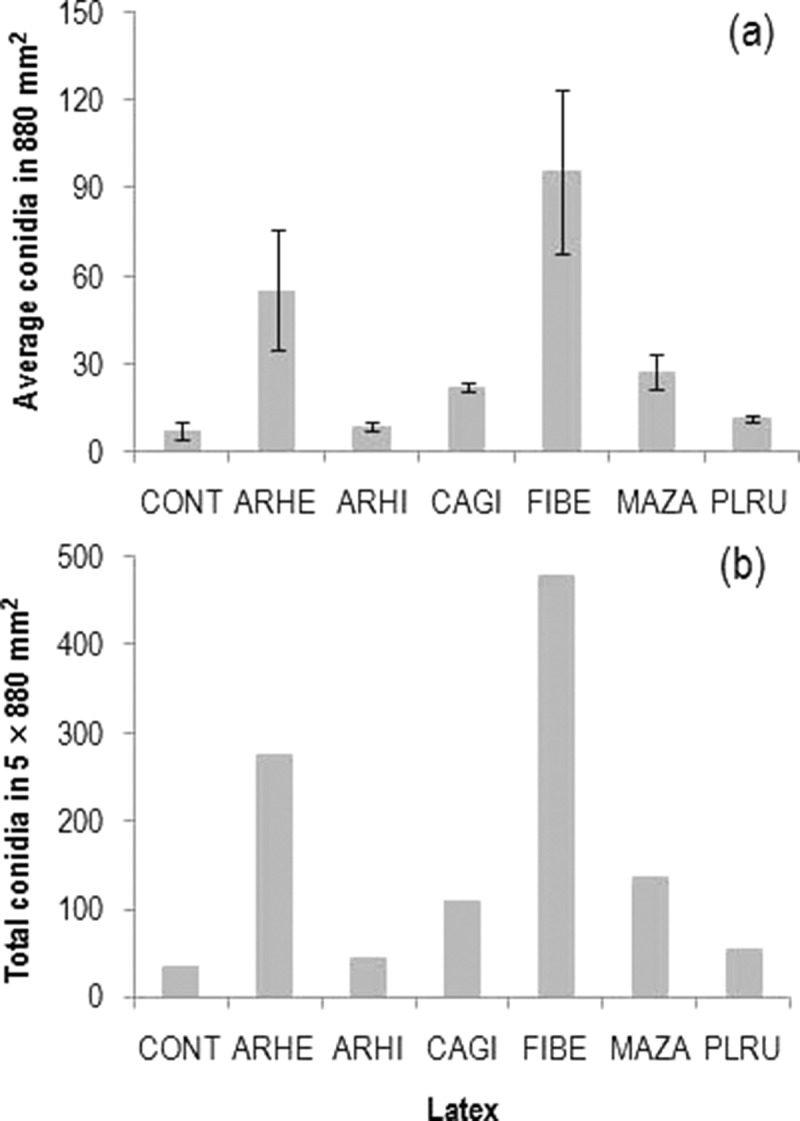
Average conidia (in 880 mm^2^) (*n* = 5, mean ± SE) (a) and total conidia (in 5 × 880 mm^2^) (b) of aquatic hyphomycetes adhered on six latexes in comparison with control (CONT) (latex code: ARHE, *Artocarpus heterophyllus*; ARHI, *A. hirsutus*; CAGI, *Calotropis gigantea*; FIBE, *Ficus benghalensis*; MAZA, *Manilkara zapota*; PLRU, *Plumeria rubra*).

**Figure 4.**
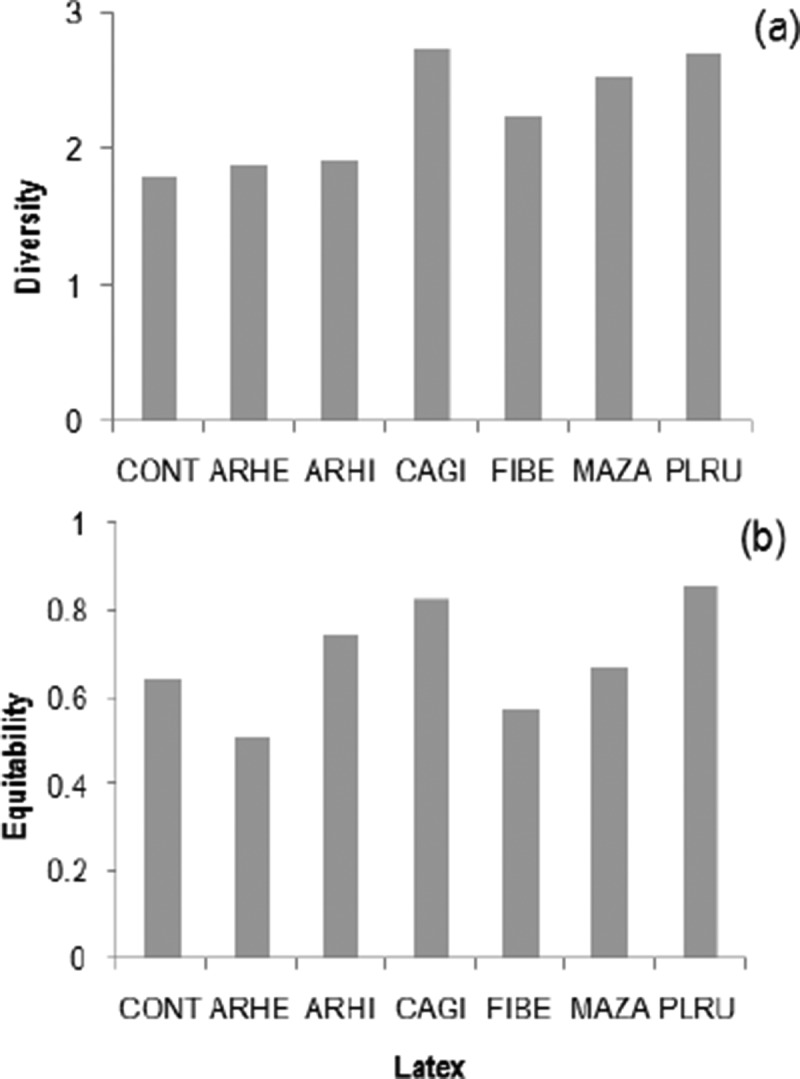
Diversity (a) and equitability (b) of aquatic hyphomycetes adhered on six latexes in comparison with control (CONT) (latex code: ARHE, *Artocarpus heterophyllus*; ARHI, *A. hirsutus*; CAGI, *Calotropis gigantea*; FIBE, *Ficus benghalensis*; MAZA, *Manilkara zapota*; PLRU, *Plumeria rubra*).

Among the latexes, *F*. *benghalensis* attracted the highest average and total number species, while it was lowest in *A*. *hirsutus* (Figure 2). The average number of species was lowest in control slides, while the total number of species was slightly higher than in the latex of *A. hirsutus* (7 vs. 6 species) (Table 3). Similar to species, the average and total conidial adherence on latex of *F. benghalensis* was the highest, while it was lowest on control slides (Figure 3). The Shannon diversity was highest in latex of *C*. *gigantea* followed by *P*. *rubra* (lowest on control slides), while the opposite was found for the equitability among these latexes (Figure 4).

### 3.4. Species richness and community similarity

Sørensen’s similarity among the latexes was highest between *F. benghalensis* and *M*. *zapota* (89.6%) and lowest between *A. hirsutus* and *P. rubra* (13.3%) (Table 4). Comparison between control and latexes revealed the highest similarity of control with *C. gigantea* (62.5%) and lowest with *P. rubra* (25%).

**Table 4.** Sørensen’s similarity (%) of aquatic hyphomycete communities from conidia adhered to control (CONT) and latex-coated slides (ARHE, *Artocarpus heterophyllus*; ARHI, *A. hirsutus*; CAGI, *Calotropis gigantea*; FIBE, *Ficus benghalensis*; MAZA, *Manilkara zapota*; PLRU, *Plumeria rubra*).

	ARHE	ARHI	CAGI	FIBE	MAZA	PLRU
CONT	52.6	61.5	62.5	54.5	57.1	25.0
	ARHE	55.5	51.6	37.0	69.2	38.0
		ARHI	53.3	57.1	45.5	13.3
			CAGI	66.6	60.8	44.4
				FIBE	89.6	50.0
					MAZA	43.4

The one-way ANOVA revealed significant differences in species richness as well as conidia attached on the six latexes comparing to control slides. Significant differences in the overall species richness were found between *F. benghalensis* and control, *A. hirsutus* and *P. rubra* (Tukey’s post-test, *P* < 0.001 to *P* < 0.05); and between control and *A. heterophyllus* (Tukey’s post-test, *P* < 0.05). In addition, average conidial richness was significantly different between *F. benghalensis* and control, *A. hirsutus* and *P. rubra* (Tukey’s post-test, *P* < 0.05, for all comparisons); and between control and *A. heterophyllus* (Tukey’s post-test, *P* < 0.05).

## 4. Discussion

Although a total of 2 l of stream water (100 ml in five sites during four occasions in 18 h duration) was filtered, the total number of species of aquatic hyphomycetes did not attain more than 10 (average species, 4.8 100 ml^−1^; average conidia, 34 100 ml^−1^). Mere exposure of a small area of latex-coated slides (880 mm^2^) of *A*. *heterophyllus* and *F*. *benghalensis* in stream supported higher species as well as conidia of aquatic hyphomycetes than water samples. The trapping efficiently of conidia by the other latexes (*A*. *hirsutus, C. gigantea, M. zapota* and *P*. *rubra*) was low possibly due to their nature of stickiness or latex chemistry. However, the Shannon diversity in latex-coated slides of *C. gigantea, M. zapota* and *P. rubra* was higher than *F. benghalensis*. The assemblage of aquatic hyphomycetes in four latex-coated slides (*A. heterophyllus, C. gigantea, F. benghalensis* and *M. Zapota*) and water is comparable as the top five species are the same (*A*. *longissima, F. curvula, L. curvula, T. monosporus* and *T. konajensis*). Similarly, the top three species (*L. curvula, T. monosporus* and *T. konajensis*) partially resembles with an earlier study in Konaje stream by monitoring water, foam and leaf litter (Sridhar et al. 1992) supporting that latex-coated slides are reliable in monitoring aquatic hyphomycete communities in streams.

Assessment of leaf litter, water and foam samples in Konaje stream yielded about 18–20 species of aquatic hyphomycetes (Sridhar and Kaveriappa 1984; Sridhar et al. 1992, 2013). Pooled data on latex-coated slides in our study showed 21 species and on the banyan (*F. benghalensis*) latex-coated slides entrapment of conidia was as highest as 15 species. Control slides and latexes of *A. hirsutus* and *P. rubra* were not as efficient as latex of banyan, which trapped the lowest number of aquatic hyphomycete species and conidia. Based on the One-way ANOVA, latex of banyan trapped significantly higher number of species and conidia of aquatic hyphomycetes than control slides as well as two latexes (*A. hirsutus* and *P. rubra*). The latex *A. heterophyllus* also entrapped significantly higher number of species and conidia than control slides. Moreover, banyan latex-coated slides seem to be not biased to trap sigmoid or multiradiate conidia as it has represented by seven and eight species, respectively. With these results, in comparison with control slides and conidial drift, our observations suggest that latex of banyan is highly useful in monitoring diversity of aquatic hyphomycetes especially in tropical streams. More studies would be needed to ascertain suitability of banyan and other latex in subtropical and temperate streams for assessment of community of aquatic hyphomycetes. Interestingly, baiting banyan leaf litter in the streams of Southwest India resulted in recovery of maximum number of species of aquatic hyphomycetes (Sridhar and Kaveriappa 1989; Sridhar et al. 1992).

The present study complements with an earlier study carried out on trapping aquatic hyphomycete conidia on rosin-coated slides by Bärlocher et al. (1977). Extensive conidial germination, formation of appressoria and mycelial growth above 18 h support that latexes are likely to serve as nutritional source. However, conidia of *T. konajensis* grew and sporulated on five submerged latexes coated on slides in the laboratory, but others such as *Ingoldiella hamata* and *T*. *acuminatus* selectively grew and sporulated on latexes indicating specificity of latex in promoting anchorage, growth and sporulation (Sridhar and Kaveriappa 1987). As reported by Bärlocher et al. (1977) on rosin-coated slides, latex-coated slides also did not attract terrestrial conidia such as *Alternaria, Drechslera* and *Fusarium*. However, a few conidia of *Tetraploa aristata* were attached to the latex of *A. heterophyllus, C. gigantea* and *F. benghalensis*). Compared to water, leaf litter and foam analysis, latex-coated slide technique is simple and economical to monitor aquatic hyphomycetes in streams. Water filtration and bubble-chamber incubation of leaf litter require microbiological filters to trap the conidia. Although foam can be assessed either directly or using filters, its occurrence is not consistent in all streams and also at the time of sampling.

This is the first study adapting latex-coated slides for entrapment and assessment of conidia of aquatic hyphomycetes in streams. Monitoring stream waters for occurrence of aquatic hyphomycetes has several advantages to understand the population dynamics in relation to a variety of perturbations and environmental stresses. Assessment of aquatic hyphomycetes in streams using latex of banyan (*F*. *benghalensis*) is a simple and cost-effective technique. Further studies are needed to assess seasonal/diurnal pattern of entrapment of conidia on latex-coated slides in streams. It is also interesting to evaluate whether latex-smears besides adhering substrate serve as nutritional source for these fungi. In addition to attachment of morphologically distinct asexual spores, latex may also attracts ascospores and basidiospores which cannot be identified as easily as aquatic hyphomycetes demanding application of molecular techniques.

## Disclosure statement

No potential conflict of interest was reported by the authors.
